# Supplementary figures and images for: An atrial fibrillation-associated regulatory region modulates cardiac Tbx5 levels and arrhythmia susceptibility
Source: eLife. 2023 Jan 30;12:e80317. doi: 10.7554/eLife.80317 (PMC9928424; doi:10.7554/eLife.80317)

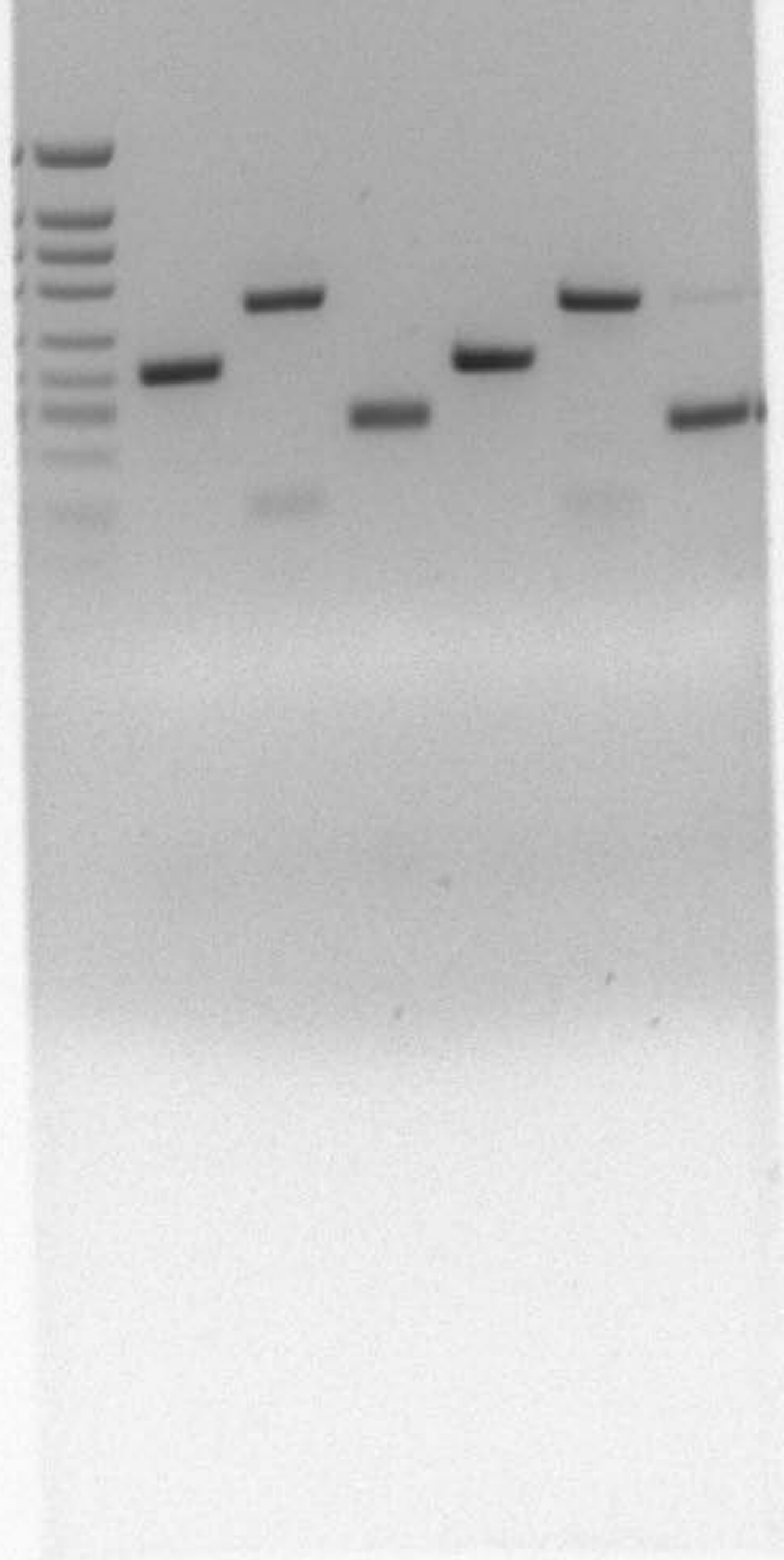

Supplement: Figure 2—figure supplement 3—source data 1. [file elife-80317-fig2-figsupp3-data1.zip › Fig2figsup3sourcegel.pdf]
